# Dynamic household structure and composition: a manual for longitudinal analysis of living arrangements

**DOI:** 10.1186/s13104-023-06485-x

**Published:** 2023-09-19

**Authors:** Ashira Menashe-Oren, Yacouba Compaoré, Philippe Bocquier, Carren Ginsburg

**Affiliations:** 1 Centre for Demographic Research, Université catholique de Louvain, Louvain-la-Neuve, Belgium; 2 Medical Research Council/Wits Rural Public Health and Health Transitions Research Unit (Agincourt), School of Public Health, Faculty of Health Sciences, University of the Witwatersrand, Johannesburg, South Africa; 3 Institut Supérieur des Sciences de la Population (ISSP), Université Joseph KI-ZERBO, Ouagadougou, Burkina Faso

**Keywords:** Longitudinal data, Household structure, Household composition, Health and demographic surveillance systems, Event history analysis, Time–varying covariates

## Abstract

**Objective:**

This research note introduces a set of tools to conduct analysis of household structure and composition with either limited or comprehensive longitudinal data. The data used here are from Health and Demographic Surveillance Systems in Africa, but the methods can be adapted and applied to other longitudinal micro-data such as register data. A training manual describing the procedures for creating time-varying household measures step-by-step is supplied as supplementary material to this note. Code is provided in STATA but can easily be translated for other statistical software, and the logic for each step remains the same.

**Results:**

The analysis of household structure demonstrates how with limited data (such as a household identifier), it is possible to construct time-varying measures of household membership, including household size or the number of members in specific age and sex groups. The analysis of household composition demonstrates how with expanded data (including links to parents in addition to residence status in the same household), it is possible to construct time-varying measures of household membership of specific kin, i.e. mother, sibling or grandparent. The results illustrated in this research note are a taste of what can be achieved by following the training manual in the supplementary material.

**Supplementary Information:**

The online version contains supplementary material available at 10.1186/s13104-023-06485-x.

## Introduction

Households are the building blocks of societies, and are an important demographic unit of analysis. One of the primary social functions of households is to care for children until they support themselves [1]. Households, and families, have been identified as important for child wellbeing and survival [2–9]. As such, social and health surveys regularly ask individuals about their living arrangements. However, such surveys generally ask about current household status, at the time of data collection, and not at the time of a child’s death, or any other event of interest. Time-varying living arrangements are especially important to consider when examining causal relationships in health outcomes.

Here we explore the changes a child may experience in her living arrangements, from birth to age five, and demonstrate that even with limited longitudinal data, complex and time-varying household covariates can be constructed. Essentially, our method consists of bringing into the biographical records of each individual, records of other people with whom this individual lived. We use Health and Demographic Surveillance Systems (HDSS) data from sub-Saharan Africa to unpack the multiple ways we can work with household data. The purpose of this Research Note is to offer an empirical approach for capturing time-varying living arrangements with covariates that can be used to model, say, mortality of children as they age, i.e. at each observation time from birth and not just at the time of birth, death, or censoring. We provide two different approaches to measure household structure and household composition, which can be employed according to the availability of data, and can be extended and adapted to exploring living arrangements of specific sub-categories of individuals (for example, the elderly).

## What is a household?

Households are commonly defined as a group of persons who share food, shelter, and other essentials for living [1, 10]. However, this definition varies considerably across countries. It is not the purpose of this article to define households, but it is important to acknowledge that they can mean different things in different contexts. For example in South Africa it is common to have household members who are not co-resident [11], challenging the very definition of household members sharing a common living space. When we refer to household we refer to how the “statistical household” was defined in data collection. Here, we do not address standardization needed for comparability or diverging concepts, nor context-specific culturally-defined households.

Importantly, the boundaries of households overlap with those of family. As such, household composition refers to the kin relations between household members. Household composition relies on the relationships between parents, children, siblings, grandparents, and other relatives. In contrast, household structure refers to the presence of individuals according to their age and sex [2]. Structure is about the presence of multiple generations, and the extent of nucleation. Nuclear household structure is the presence of two adults (commonly one male and one female) of working-ages and children, while nuclear household composition is the presence of two married or cohabitating partners, and their children.

## Methods and data

HDSS collect longitudinal data on populations within low- and middle-income countries where vital registration is lacking. The HDSS monitors population events within a geographically delimited population, ranging in size from a couple of thousand individuals to over 100,000. During a baseline census, each household within the surveillance area is visited, and detailed information gathered on each household member [12]. Following this initial data collection, each household is then visited at least once a year, updating key information,^1^ and tracking each household – whether it splits, leaves the site or relocates locally. Key events recorded for each individual are their birth, death, and in- or out-migration from the HDSS site, including the precise time of these events [12]. Each individual in the HDSS is recorded with a unique identifier (ID), and connected to a household ID. Individuals keep their ID throughout their lives (even if they out-migrate and then return), but may change household IDs (which do not only link members, but are also related to a location within the site). HDSS data are rigorously collected, detailed and with chronological documentation [13], and can therefore be used for event history analysis.

Standardised and readily-available HDSS data are accessible through the International Network for the Demographic Evaluation of Populations and their Health (INDEPTH) iShare repsoitory [14]. We use the INDEPTH iShare data from an HDSS in The Gambia, to demonstrate household structure measures. Farafenni is a rural HDSS with surveillance commencing in 1990. To demonstrate household composition measures we also use HDSS data, obtained directly from the same site in The Gambia, Farafenni, since these data include family ties (like the identity of the father), which are not available in the INDEPTH iShare repository. The data are processed in STATA (and the code in supplementary material is provided for STATA users), but the same step-by-step logic in the manual can be employed using other statistical programmes.

### Kinship

Where the HDSS data include Mother ID (as in the INDEPTH iShare data), and Father ID (included in the data we use from The Gambia), we can identify the family ties between a child and multiple individuals within the HDSS (often within the same household). Mother’s IDs are typically most accurate, since pregnancies are followed up during HDSS surveillance. Apart from the obvious link to the parents when we have mother’s and father’s identifiers, we can also identify the child’s (Ego) siblings, as they share the same parents. Then, because we have the mother and father identifiers for Ego’s parents, we can locate the grandparents of Ego. We can further identify the siblings of the parents, who are the aunts and uncles of the child. Also, because these individuals are linked to their children, it is possible to locate the cousins of Ego. Thus, entire extended families of a child can be identified and their events matched to those of the child, if they are attached to an HDSS household (see Fig. 1 in supplementary material).

### Co-residence

**Fig. 1:**
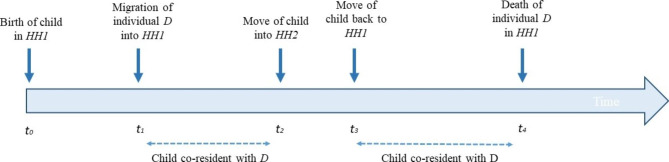
Determining co-residence of child with other individuals Note: HH stands for household, D for individual, t for time

While the kinship relations are possible to identify through the mother’s and father’s IDs, further information is needed to determine if they are living together. We can determine co-residence of the child with other individuals (whether identified as kin or not) based on the household ID, when the definition of household membership is de facto.^2^ In Fig. 1 we illustrate how co-residence is time-varying, and determined by the events of both the child and other individuals. Over the period of surveillance, the child in Fig. 1 lived with individual *D* for two periods of time, between t1 and t2, and between t3 and t4, prior to individual *D*’s death. If a survey was administered to collect data from the household where this child lives after t4, it would not account for the earlier presence of individual *D*. Yet, his death may have affected the child, after having lived together for some time.

### Measuring household structure

To measure the number of people in a household at a given time, based on the population balancing equation we use the following equation (Eq. 1):

$$HS = \sum {EN{U_{m,f|a}}} + BT{H_{m,f|a}} + IM{G_{m,f|a}} + EN{T_{m,f|a}} - DT{H_{m,f|a}} - OM{G_{m,f|a}} - EX{T_{m,f|a}}$$

Where *HS* indicates household size, *m* denotes male, *f* female and *a* age group. Events are noted as: *ENU* enumeration, *BTH* birth, *IMG* in-migration into the household from out of the HDSS site, and *ENT* indicates entry of an individual into the household, moving within the site. *DTH* indicates death, *OMG* out-migration from the household and HDSS site and *EXT* indicates an individual leaving the household but moving within the boundaries of the HDSS. The balance of events allows us to calculate the household size, as well as the number of members by age and sex at any point in time. The members can then be combined into different groups of living arrangements that reflect “typical” households. For example, a household with an identified male between ages 15–49, and a female aged 15–49, and two under-five year olds is parallel to a nuclear household.

### Measuring household composition

To measure household composition, kin relations between individuals are needed. As mentioned above, it is possible to rely on the identification of parents of all individuals to build a kin network. Kins’ event histories can be linked to the child’s (Ego) event history by merging data according to their order in time (command *tmerge* in Stata) [15]. Detailed steps of how the mother’s events (and sibling’s events) are merged with the child’s events are provided in the supplementary material. There, we also explain how to program indicators of time-varying household structure.

## Results

The following results illustrate descriptive findings at the aggregate level using time-varying covariates created at the individual level. More complex modelling results at individual level are also possible (see Menashe-Oren et al. 2023 for example [16]). Figure 2 of the supplementary material provides an illustration at the individual level.

### Household structure

**Fig. 2:**
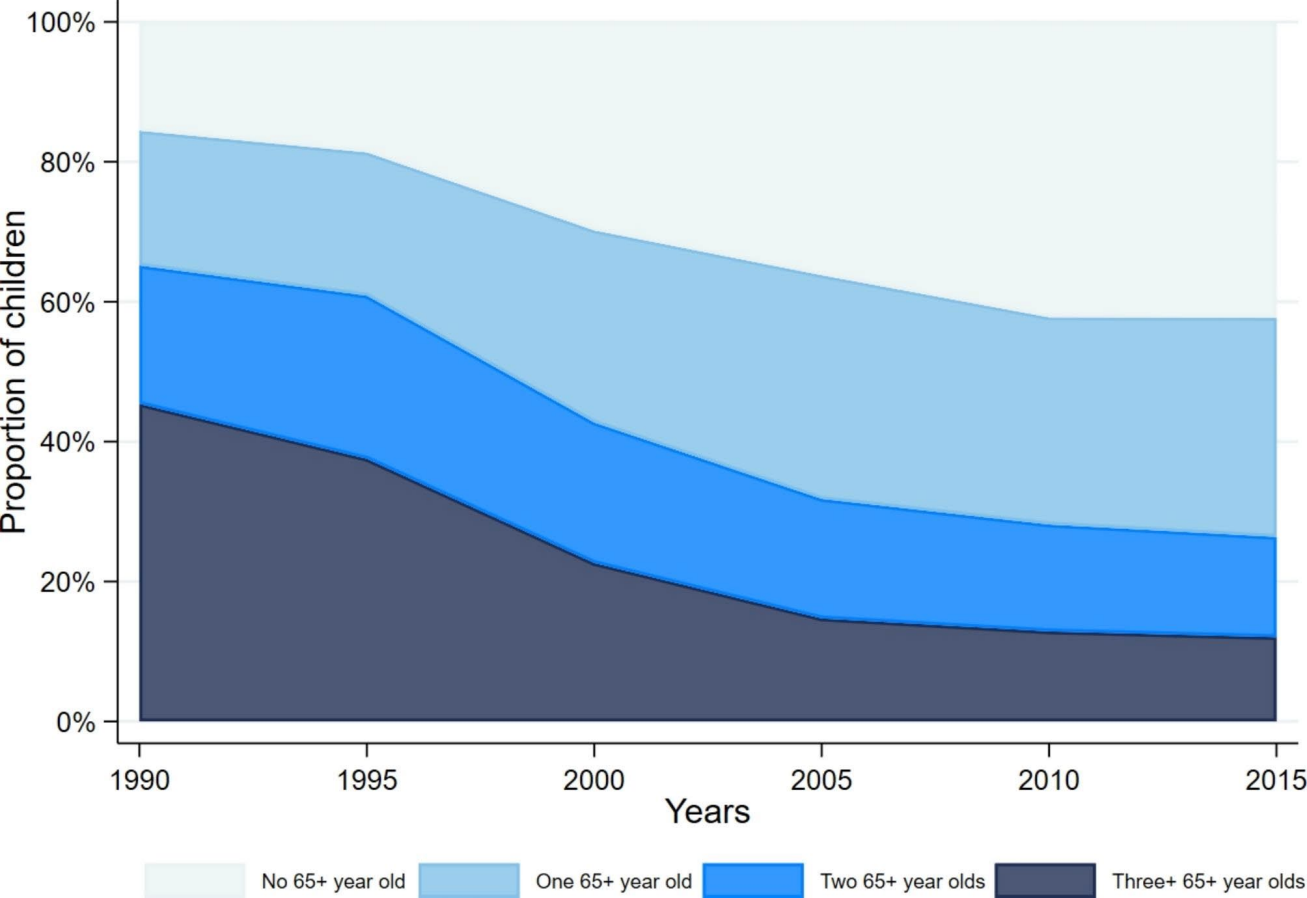
Proportion of under-five year old children living with older adults (aged 65+) over time in Farafenni HDSS

We consider the household structure, where kin relations are not provided (except for mother’s ID for children born in the HDSS), by examining the number of over 65 year-old adults with whom children under age five reside.^3^ We find that in Farafenni, there was a decline in the proportion of children living with multiple older adults (Fig. 2). In the 1990s the majority of children lived with three or more 65+ year old adults, but over time this has declined. In 2015 around 40% of children do not live with any older adults. Figure 2 illustrates time-varying counts of older aged household members, proxying grandparents, but this could easily be employed for other age groups too.

### Household composition

**Fig. 3:**
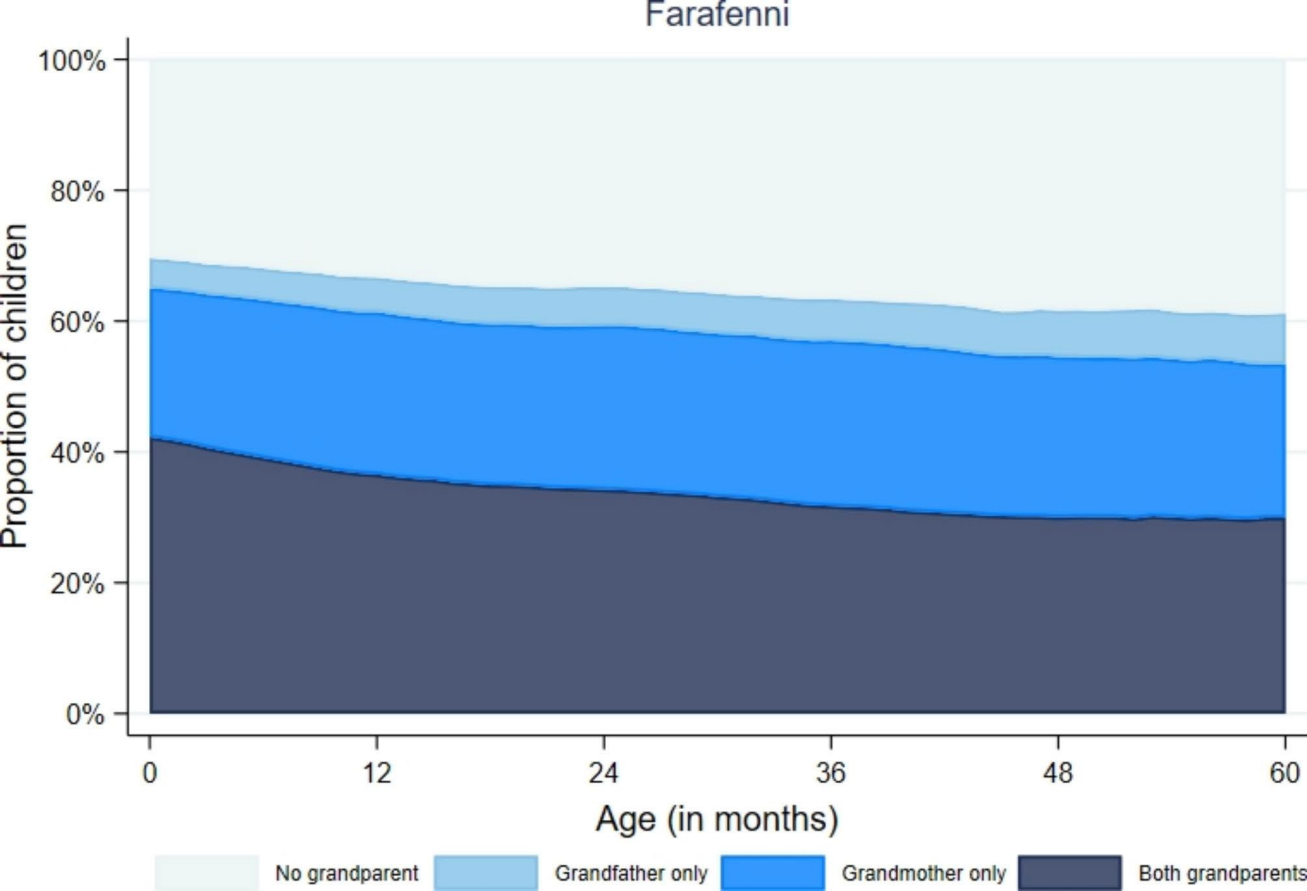
Proportion of under-five year old children living with grandparents by age of child, in Farafenni HDSS

When considering household composition based on kin relations (where it is possible to identify in the data who the mother and father are), a more accurate picture of household composition is given. Beyond identifying how many older adults aged 65 or more are in the household, we can ascertain whether they are grandparents (and even distinguish between maternal or paternal grandparents). Figure 3 illustrates how the proportion of children living with both grandparents steadily declines as the child grows up (until age five), while the proportion of children living without any grandparent increases slightly. Moreover, the proportion of children living with grandfathers only is smaller than those living with grandmothers only. This may reflect a practice of grandmothers (often widowed, since women tend to survive to older ages than men), living with one of their children, and helping to care for their grandchildren.

## Conclusion and limitations

Constructing measures of time-varying living arrangements is appealing since it allows us to examine the effect of households, and the presence of kin, on events of interest around the time of the event rather than at the time of data collection (as in surveys or census). For instance, it is possible to consider the effect of the presence of elderly in the household on the survival of children, knowing that a grandparent did not move into the household after a child’s death (and thus excluding reverse causality in analysis). Nevertheless, there are some limitations with the use of time-varying household structure and composition measures. Firstly, the data need to have records of kin relations – information which is not always collected. Without parent identifiers at a minimum, it is not possible to link kin or household members to individuals (in our case, children). All the same, we show that if we have household identifiers, we can attach individuals to households, and extract information on household structure with relative ease. Secondly, this method relies on the de facto residence of family and household members. Using a different household definition, as de jure membership, can change the interpretation (see supplementary material for further discussion of this limitation). Finally, it is important for the longitudinal data to be consistent, in the precise timing and order of events. Inaccuracy in the data can result in, for example, a negative household size.

### Electronic supplementary material

Below is the link to the electronic supplementary material.

Supplementary Material 1

## Data Availability

HDSS data are available at: http://www.indepth-ishare.org/index.php/home.

## Abbreviations

HDSS: Health and Demographic Surveillance Systems
INDEPTH: International Network for the Demographic Evaluation of Populations and Their Health

## Footnotes

1 Depending on funding, some HDSS collect data and update the baseline more frequently, sometimes even every three months. More frequent rounds allow for capturing events more accurately, particularly with regards to pregnancies and their outcomes.

2 De jure residence records individuals in a geographical area according to their regular place of residence even if at the time of the query indivduals are physically absent from the household, while de facto residence records individuals who are physically present in a geographical area at a specified time.

3 We use a 65 year- old age cut-off since the population generally becomes economically dependent from this age, and have poorer health, meaning they are in competition for resources with young children (we also akin this age group to grandparents).
