# Supplementary material for: Dynamic household structure and composition: a manual for longitudinal analysis of living arrangements
Source: BMC Res Notes. 2023 Sep 19;16:223. doi: 10.1186/s13104-023-06485-x (PMC10510116; doi:10.1186/s13104-023-06485-x)
Supplement: Supplementary file 1 — Supplementary Material 1 [file 13104_2023_6485_MOESM1_ESM.docx]

Ever-changing household structure and composition: A manual for longitudinal analysis of living arrangements

Ashira Menashe-Oren, Yacouba Compaoré, Philippe Bocquier, and Carren Ginsburg

Table of Contents

[**Introduction** 3](#_Toc142904248)

[Preparation of the core data files 3](#_Toc142904249)

[**Household structure: Linking individuals to households** 4](#_Toc142904250)

[Constructing household size and typology without any data on kinship 5](#_Toc142904251)

[Stata code for steps in creating time-varying household size 8](#_Toc142904252)

[Step 1. Create a variable identifying the first date recorded in each household 8](#_Toc142904253)

[Step 2. Count the number of each type of event in the household (excluding enumerations) 8](#_Toc142904254)

[Step 3. Count the number of enumerations in the household 9](#_Toc142904255)

[**Kinship and household composition: Linking individuals to other individuals** 12](#_Toc142904256)

[Stata code for merging mother’s events with child’s events in correct time order 14](#_Toc142904257)

[Step 1: Add a right-censoring date to the child file 14](#_Toc142904258)

[Step 2: Create for each child a file with all the events experienced by parents 16](#_Toc142904259)

[Step 3: Merge mother’s events with child’s events 21](#_Toc142904260)

[Stata code for merging child events with siblings’ events 22](#_Toc142904261)

[Step 1: Define the sibling’s true rank 22](#_Toc142904262)

[Step 2: Create files for the twin sibling, and the younger and older siblings 24](#_Toc142904263)

[Step 3: Merge the younger and older sibling files with the child’s file 27](#_Toc142904264)

[Step 4: Optional: Restricting observations to under-5 year old 28](#_Toc142904265)

[**Caveats and limitations** 29](#_Toc142904266)

[Caveats related to HDSS data collection 29](#_Toc142904267)

[Limitations related to the methodology 29](#_Toc142904268)

# **Introduction**

The supplementary material for this manual gives detailed steps to the data management and programming needed to, 1) construct household size and typology without any data on kinship, and 2) to construct household composition (with data on kinship) by linkage of a parent’s event history to ego. This manual provides a checklist needed to create a core residency file, but does not present the detailed data management and analysis steps which have already been provided in previous manuals [1, 2]. The proposed procedures can be implemented across different sources of longitudinal data including Health and Demographic Surveys (HDSS), register data, retrospective or panel survey data. In this manual we use HDSS data which clearly defines the entry and exit of individuals into and out of populations, available from the International Network for the Demographic Evaluation of Populations and Their Health (INDEPTH) iShare platform [3]. For other sources of data some changes in the programming may be needed.

# Preparation of the core data files

Here we provide a check-list to ensure the data are suitably prepared for the construction of time-varying measures of household structure and composition. Detailed instructions on the creation of a standardised file formatted for event history analysis with each individual having a unique identifier (ID) and each individual’s event recorded as a single record (a core residence data file), can be found in earlier manuals [1, 2].

1. Ensure all individuals have a first event that defines their entry in the population (such as enumeration, birth, or in-migration), and events that define their exit from the population (such as death, out-migration or right-censoring i.e. end of observation). These events define the temporal boundaries of the individual under observation. Note that there can be several entry and exit events from a population (through in- and out-migration).
2. Each event should be associated with a date. An individual’s events should be ordered in time, and not occur at the exact same time. To facilitate this, time should be stored in milliseconds (%tc format in Stata), with some hours inserted between events occurring on the same day. For example, all births may be set at mid-day, while out-migrations at 18:00, which will determine the order of these events if they occur on the same day.
3. An individual’s events need to be consistent. For example, a birth cannot follow a migration event, or there should be no events after a death, or two sequential in-migrations are not allowed. A consistency matrix can be used to identify inconsistencies in the order of events.
4. A variable defining the period of exposure in the population under study is needed for all event history analysis. This binary variable, “residence”, depends on the logical sequence of events, and relies on a time criterion for residence (such as a minimum duration of six months residence).

In addition to this check-list, it is important to have a variable for household membership (household ID) to enable the analysis of household structure, as well as a variable of parental identification (mother and/or father ID) for household composition.

# **Household structure: Linking individuals to households**

Each individual in the HDSS is recorded with a unique identifying ID, and connected to a household (called Location ID in the INDEPTH data), also given an ID. In Table S1 for example, individual KE001 is located in household HH109. KE001 was born in 2005, and at the age of seven, she in-migrated to the HDSS. She then moved to a new household (HH874), and then in 2014 out-migrated from the site. From these data we can follow the life of this individual while within the HDSS, and can place her in two different households. We can see from the household ID that individual KE035 is in the same household as KE762. The boy KE035 is actually the son of KE762 (based on the Mother ID in Table S1), and his mother died almost two months after she gave birth to him. Thus, through the Mother ID we can link the child’s events with the mother’s events (as we show later in the manual).

Table S1: Example of HDSS data for three individuals from a site in Kenya

| **Individual ID** | **Household ID** | **Event Type** | **Event Date** | **Date of Birth** | **Sex** | **Mother ID** |
| --- | --- | --- | --- | --- | --- | --- |
| KE001 | HH109 | IMG | 15/02/2012 | 23/04/2005 | Female |  |
| KE001 | HH109 | EXT | 19/04/2013 | 23/04/2005 | Female |  |
| KE001 | HH874 | ENT | 20/04/2013 | 23/04/2005 | Female |  |
| KE001 | HH874 | OMG | 20/05/2014 | 23/04/2005 | Female |  |
| KE001 | HH874 | OBE | 31/12/2015 | 23/04/2005 | Female |  |
| KE035 | HH023 | BTH | 03/10/2015 | 03/10/2015 | Male | KE762 |
| KE035 | HH023 | OBE | 31/12/2015 | 03/10/2015 | Male | KE762 |
| KE762 | HH023 | ENU | 31/12/2002 | 14/07/1979 | Female |  |
| KE762 | HH023 | DTH | 24/11/2015 | 14/07/1979 | Female |  |
| KE762 | HH023 | OBE | 31/12/2015 | 14/07/1979 | Female |  |

^Note: ENU= enumeration, IMG=in-migration, OMG=out-migration, BTH=birth, ENT=enter new household, EXT= exit household, DTH=death, OBE=end of observation^

Time-varying household structure can be captured by measures of household size, dependency ratios, sex ratios, the number of members in specific age and sex groups, or classified along a household typology. For example, the household size is two for HH023 in Table S1 between the birth of individual KE035 until individual KE762 (his mother) dies, the sex ratio is one, and the dependency ratio is one. The same household could also be classified as a “single mother” household by considering the number of members in each age and sex group (in this case, an adult woman, and a young child).

## Constructing household size and typology without any data on kinship

The data needed to construct household measures (size) and membership of specific age and sex categories (on which it is possible to build sex ratios, dependency ratios, or a typology of households), are individual identifier codes (IDs, akin to names), household identifiers, sex of individual, date of birth of individual, type of event recorded for the individual that will include/exclude her from the population (e.g. birth, migration), and the date of this event. In Table S2 an example of the data needed to construct household size is given for one household of four individuals.

Table S2: Example of HDSS data for four individuals connected to one household, and the data needed to construct household size

| Individual ID | Household ID | Sex | Event Code | Event Date | Date of Birth |
| --- | --- | --- | --- | --- | --- |
| KE64 | KE100811 | Female | 3. IMG | 14/03/2010 | 12/03/2009 |
| KE64 | KE100811 | Female | 5. EXT | 09/03/2011 | 12/03/2009 |
| KE64 | KE102374 | Female | 6. ENT | 10/03/2011 | 12/03/2009 |
| KE64 | KE102374 | Female | 9. OBE | 31/12/2015 | 12/03/2009 |
| KE06 | KE100811 | Female | 1. ENU | 31/12/2002 | 14/07/1974 |
| KE06 | KE100811 | Female | 4. OMG | 15/05/2003 | 14/07/1974 |
| KE06 | KE100811 | Female | 9. OBE | 31/12/2015 | 14/07/1974 |
| KE132 | KE100811 | Male | 1. ENU | 31/12/2002 | 30/10/1938 |
| KE132 | KE100811 | Male | 7. DTH | 20/04/2015 | 30/10/1938 |
| KE132 | KE100811 | Male | 9. OBE | 31/12/2015 | 30/10/1938 |
| KE502 | KE100811 | Female | 2. BTH | 14/07/2012 | 14/07/2012 |
| KE502 | KE100811 | Female | 9. OBE | 31/12/2015 | 14/07/2012 |

^Note: ENU= enumeration, BTH= birth, IMG= in-migration, OMG= out-migration, EXT= exit household (moving within HDSS site), ENT= enter household (within HDSS), DTH= death, OBE= end of observation^

To construct the time-varying household size, the data need to be sorted by Household ID and Event Date. Then each event type is counted, and summed up, as demonstrated in Table S2. Before this is done, it is important to ensure that the data are prepared correctly as outlined above. In particular, the sequence of events of each individual should make sense. The sequence of events should make sense at the household level too. For example, a household’s first event cannot be an out-migration. An enumeration (or birth) should precede this. Otherwise, there will be cases of negative household size. As we can see in Table S3, the household size changes over time. The household size at time of the child’s birth in comparison to the end of observation period in the HDSS (time of right-censorship) is different.

Table S3: Computing household size based on example of individuals in Table S1

| Individual ID | Household ID | Event Code | Event Date | Enu | Total Enu | Bth | Total Bth | Img | Total Img | Ent | Total Ent | Dth | Total Dth | Omg | Total Omg | Ext | Total Ext | HH size |
| --- | --- | --- | --- | --- | --- | --- | --- | --- | --- | --- | --- | --- | --- | --- | --- | --- | --- | --- |
| KE06 | KE100811 | 1. ENU | 31/12/2002 | 1 | 0 |  |  |  |  |  |  |  |  |  |  |  |  | 0 |
| KE132 | KE100811 | 1. ENU | 31/12/2002 | 1 | 0 |  |  |  |  |  |  |  |  |  |  |  |  | 0 |
| KE64 | KE100811 | 3. IMG | 14/03/2010 |  | 2 |  |  | 1 | 0 |  |  |  |  |  |  |  |  | 2 |
| KE64 | KE100811 | 5. EXT | 09/03/2011 |  | 2 |  |  |  | 1 |  |  |  |  |  |  | 1 | 0 | 3 |
| KE502 | KE100811 | 2. BTH | 14/07/2012 |  | 2 | 1 | 0 |  | 1 |  |  |  |  |  |  |  | 1 | 2 |
| KE06 | KE100811 | 4. OMG | 15/05/2013 |  | 2 |  | 1 |  | 1 |  |  |  |  | 1 | 0 |  | 1 | 3 |
| KE132 | KE100811 | 7. DTH | 20/04/2015 |  | 2 |  | 1 |  | 1 |  |  | 1 | 0 |  | 1 |  | 1 | 2 |
| KE06 | KE100811 | 9. OBE | 31/12/2015 |  | 2 |  | 1 |  | 1 |  |  |  | 1 |  | 1 |  | 1 | 1 |
| KE132 | KE100811 | 9. OBE | 31/12/2015 |  | 2 |  | 1 |  | 1 |  |  |  | 1 |  | 1 |  | 1 | 1 |
| KE502 | KE100811 | 9. OBE | 31/12/2015 |  | 2 |  | 1 |  | 1 |  |  |  | 1 |  | 1 |  | 1 | 1 |
| KE64 | KE102374 | 6. ENT | 10/03/2011 |  |  |  |  |  |  |  |  |  |  |  |  |  |  |  |
| KE64 | KE102374 | 9. OBE | 31/12/2015 |  |  |  |  |  |  |  |  |  |  |  |  |  |  |  |

^Note: ENU= enumeration, BTH= birth, IMG= in-migration, OMG= out-migration, EXT= exit household (moving within HDSS site), ENT= enter household (within HDSS), DTH= death, OBE= end of observation, HH= household^

It is important to note that household size is computed to reflect the size at the time of the event (a moment before the event takes place), rather than after the event. It therefore is not included in the estimation of size at the time of event, and is coded as zero in the “Total” columns. In other words, the total size is lagged, and included in the following event. Moreover, individual KE64 leaves household KE100811, and moves to a different one, KE102374. In Table S3 we do not show all the events and individuals associated with the new household and so the last two lines in Table S3 are left blank. These lines would be integrated into the sequence of events in household KE102374, but for simplicity we only show results from one household.

## Stata code for steps in creating time-varying household size

###

### Step 1. Create a variable identifying the first date recorded in each household

Note, the file used here is named residency, referring to a core residence datafile that has already been prepared according to a previous manual on event history analysis [1].

use residency.dta, clear

bys HouseholdId (EventDate EventCode): gen firsts=1 if EventDate==EventDate[1]

### Step 2. Count the number of each type of event in the household (excluding enumerations)

The same code can be repeated for all events except for enumeration which is slightly different. Below is an example of counting all the out-migrations from a household. EventCode is coded as: 1- ENU (enumeration), 2-BTH (birth), 3-IMG (in-migration), 4-OMG (out-migration), 5-EXT (exit), 6-ENT (entry), 7-DTH (death), 9-OBE (end of observation).

First, identify the out-migration events in a new variable:

sort HouseholdId EventDate EventCode

gen out=EventCode==4

replace out=. if out==0

To count the cumulative sum of out-migrations (as time-varying).

capture drop total_out

sort HouseholdId EventDate IndividualId

bys HouseholdId (EventDate IndividualId): gen temp=sum(out==1)

egen total_out = max(temp), by(HouseholdId EventDate)

Note that the count should be after the event and not at the time of the event (out-migration). For example, if there were two out-migrations, at the time of the second out-migration we record only one out-migration. At the time of an event following the second out-migration, we record two out-migrations.

qui sum temp

local mx = r(max)

forval lmt=0/`mx'{

replace total_out=`lmt' if out==1 & temp[_n-1]==`lmt'

}

drop temp

An adjustment for out-migrations from the same household on the same date is needed.

replace total_out=total_out[_n-1] if out==1 & out[_n-1]==1 & EventDate[_n]==EventDate[_n-1]

####

In case of inconsistent data (where out-migration is a first event in the household), reset the total to zero (as done with enumeration)

replace total_out=0 if firsts==1

###

The same codes apply to in-migration (IMG), death (DTH), birth (BTH), exit (EXT) and entry (ENT).

### Step 3. Count the number of enumerations in the household

Identify the enumeration event, and count the cumulative sum of enumerations:

sort IndividualId EventDate EventCode

gen enum=EventCode==1

replace enum=. if enum==0

capture drop total_enum

sort HouseholdId EventDate IndividualId

bys HouseholdId (EventDate IndividualId): gen temp=sum(enum==1)

egen total_enum = max(temp), by(HouseholdId EventDate)

Replace household size with zero at time of enumeration:

replace total_enum=0 if enum==1

drop temp

Finally, when we use the population balancing equation, and add up all the events that include individuals in a household, and subtract the events that exclude them (see Eq. 1 in main text), we get the household size.

gen hh_size = total_enum + total_bth + total_inm + total_ent - total_out - total_dth - total_ext

Although four individuals are connected to Household ID KE100811, at no time do all four live in the same house at the same time. Following enumeration there are two individuals in the household, and then another in-migrates making it three. The in-migrant then moves to a different household on site, so that at time of birth of the child, there are again only two other individuals in the household. Then following a death and out-migration, at the end of the observation period (end of 2015), there is only one individual in the site (see Table S2).

The same code to create household size can be looped over for specific age and sex groups. To do this, a required earlier step is to split time into the age groups of interest (for example five-year age groups). The code for this has been outlined in the earlier manual on event history data management [1]. After splitting by age, and recomputing the censoring variables after the split, it is possible to loop over age (in the below example the six age groups are coded as 0 to 5) and sex groups, and count the number of household members in each group. Note, it is not necessary to loop over birth events for all age groups, but only for the youngest age group (in our case, under-five year olds).

forval ag=0/5 {

forval sex=1/2 {

sort HouseholdId EventDate EventCode

gen x`sex'age`ag'_4=EventCode==4 & group_age==`ag' & gender==`sex'

replace x`sex'age`ag'_4=. if x`sex'age`ag'_4==0

[… *see code in step 2*…]

}

}

In this way, we can create time-changing variables such as those presented in the article, of children under age five living with 65+ year-olds. While this is at the aggregate level, it is possible to consider the trajectory of each child, and whether she lives with an older-aged adult or not at each age. We provide an illustration of such individual-level analysis in Figure 1, where child 1 lives with three over 65 year-olds until her death at age 3, while child 2 lives with two over 65 year-olds until age two and then only one older adult, child 3 lives in a household without any over-65 year old adult until age five, and child 4 in-migrates at age 3 (beginning surveillance), and lives with one older adult. Such individual-level analysis has been used to study the role of living arrangements in under-five mortality [4].

Figure 1: The number of over 65 year-olds co-residing with children (from birth to age 5): examples of time-varying living arrangements


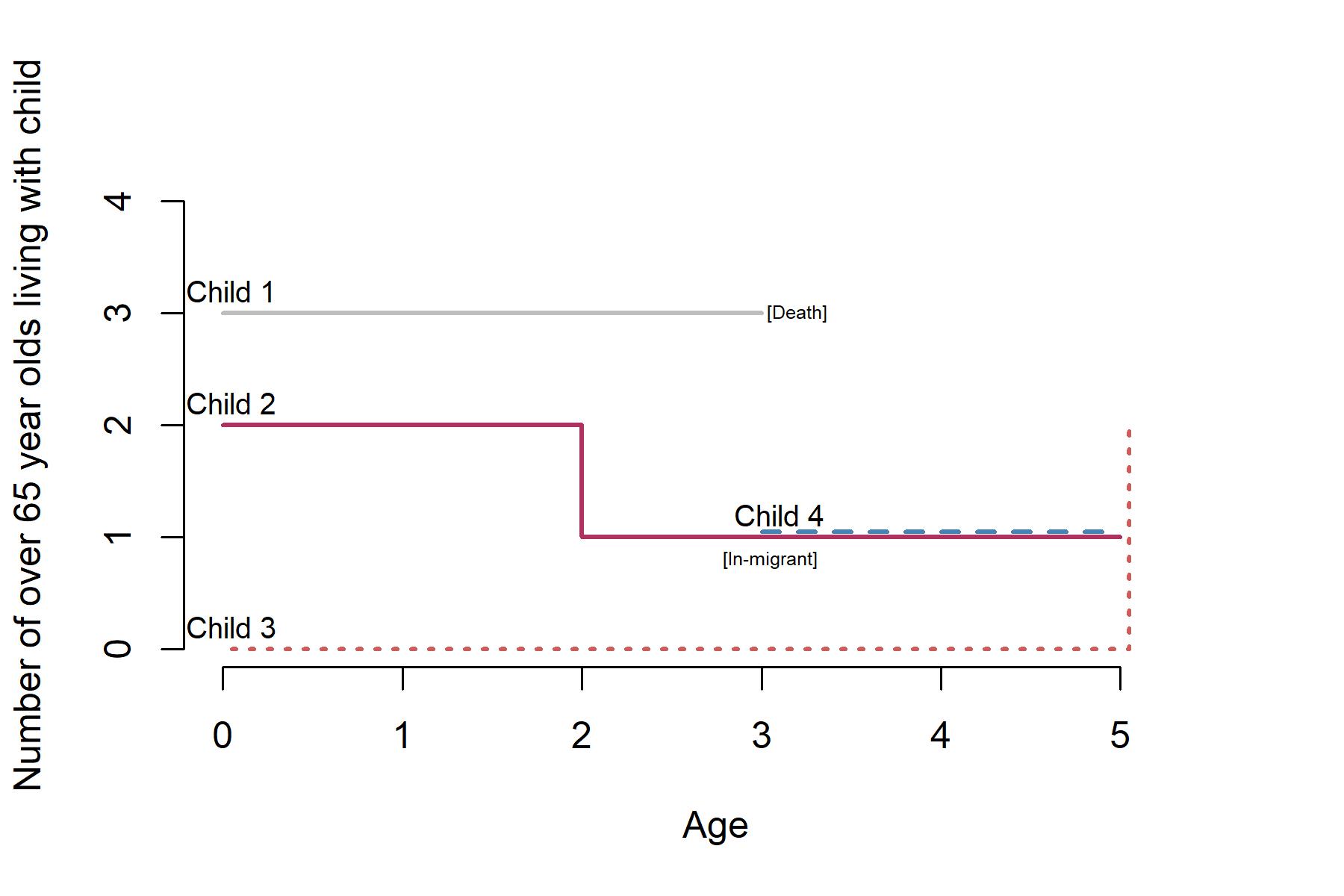


# **Kinship and household composition: Linking individuals to other individuals**

Where the HDSS data include Mother ID (as in Table S1, and in the INDEPTH data), and Father ID (as in the data we use from Senegal), we can identify the family ties between a child and multiple individuals within the HDSS (often in the same household). In Figure 2 we map out the potential kin relations we can identify between the child (“Ego”) and others. Apart from the obvious link to the parents when we have mother’s and father’s identifiers, we can also identify Ego’s siblings, as they share the same parents (see [5] and <https://github.com/bocquier/mighealth> for this Stata code). Then, because for Ego’s parents we also have the mother and father identifiers, we can locate the grandparents of Ego. We can further identify the siblings of the parents, who are the aunts and uncles of the child. And, because these individuals are also linked to their children, it is possible to locate the cousins of Ego. Thus, entire extended families of Ego can be identified if family members are present in the HDSS, and their events matched to those of Ego.

Figure 2: Determining kin relations with Ego

***
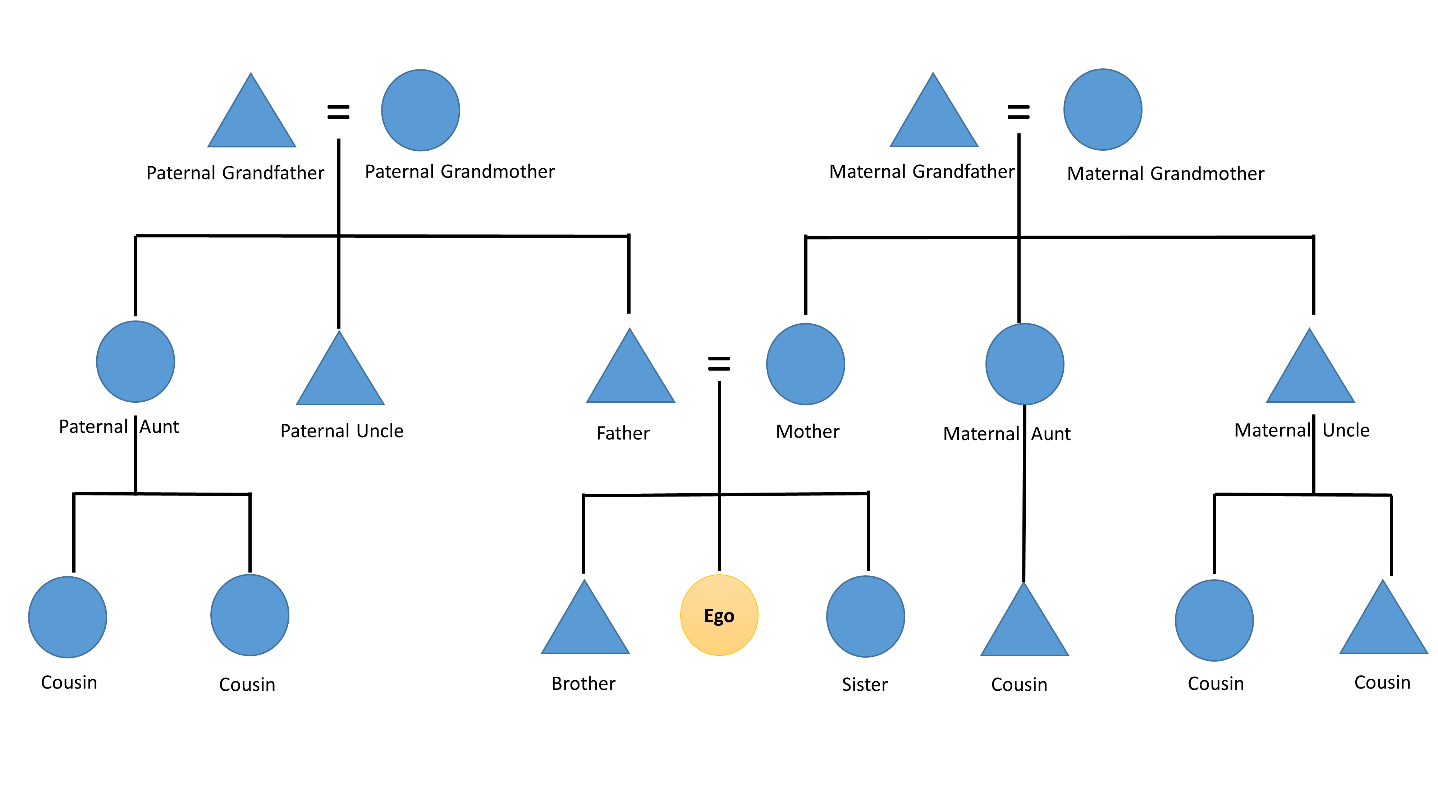
***

^Note: Triangles symbolize males, circles females, and = partnering^

Family member’s event histories can be linked to Ego’s event history. This is done using tmerge in Stata, which merges the events of the individuals according to time [1]. In Table S4 we provide an example of what the merged event histories would look like, using the same individuals as in Table 1. From the point of view of Ego, we can now see that the child’s mother died roughly two months after his birth.

Table S4: Example of linked individuals’ event histories after merging of events according to time

| Household ID | Individual ID | Child Event Type | Mother Event Type | Event Date | Date of Birth | Sex | Mother ID |
| --- | --- | --- | --- | --- | --- | --- | --- |
| HH023 | KE035 | (Mother's event) | ENU | 31/12/2002 | 03/10/2015 | Male | KE762 |
| HH023 | KE035 | BTH | (Child's event) | 03/10/2015 | 03/10/2015 | Male | KE762 |
| HH023 | KE035 | (Mother's event) | DTH | 24/11/2015 | 03/10/2015 | Male | KE762 |
| HH023 | KE035 | OBE | OBE | 31/12/2015 | 03/10/2015 | Male | KE762 |

Detailed steps of how the mother’s events (and father’s and sibling’s events) are merged with the child’s events are provided below. It is necessary to create a file for each child with all events experienced by parents. This is possible using the mother and father’s identification numbers (MotherId and FatherId). The steps provided below use mothers as an example, but can be replicated for fathers (and other relatives too such as partners, aunts and uncles, siblings, or any other family member). To merge the child and parent’s events, a number of files need to be created along the way.

## Stata code for merging mother’s events with child’s events in correct time order

As above, this code works with a core residency file (residency), after following data preparation and management steps outlined in an earlier manual [2]. Several steps are necessary to generate a child_mother_partner file:

- Step 1: Add a right-censoring date to the child file
- Step 2: Create for each child a file with all the events experienced by the mother
- Step 3: Merge mothers’ events with child’s events

### Step 1: Add a right-censoring date to the child file

In order to combine events from different individuals, an important first step is to ensure that all individuals are right-censored at the same time in the different files to merge. This is essential for using the *tmerge* command in Stata [2]. The left-censoring date can be easily handled at the analysis stage. The right-censoring date is better handled at the data management stage. Adding a right-censoring date means in practice adding an extra record for an OBE (in the example below, 01 Jan 2015).

For all individuals who died or out-migrated before 1 Jan 2015 00:00:00, the procedure is the following:

use residency, clear

sort IndividualId EventDate EventCode

expand=2 if IndividualId!=IndividualId[_n+1] ///

& EventDate< clock("01Jan 2015","DMY"), gen(duplicate)

sort IndividualId EventDate EventCode duplicate

quietly by IndividualId: ///

replace EventDate=clock("01Jan 2015","DMY") if duplicate==1

drop duplicate

For all individuals who survived as resident after 1 Jan 2015 00:00:00 and who potentially experienced events after that date, a new record must also be created:

capture drop lastrecord

quietly by IndividualId: gen lastrecord=(_n==_N)

stset EventDate, id(IndividualId) failure(lastrecord==1) exit(time .)

capture drop calendar

stsplit calendar, at(clock("01Jan 2015","DMY")) trim

drop lastrecord

drop _*

Drop the unnecessary observations after 1 Jan 2015 00:00:00 (the right-censoring date) and possible duplicates:

drop if calendar!=0

duplicate drop

For all individuals the last, right-censoring record needs to be recoded as an OBE (coded 9):

sort IndividualId EventDate EventCode

quietly by IndividualId : replace EventCode=9 if _n==_N

quietly by IndividualId : replace EventDate= clock("01Jan 2015","DMY")if _n==_N

It is always safe to check event order inconsistencies using the event consistency matrix (see first part of the manual on event history data management [2]) :

capture drop foll_EventCode

bysort IndividualId (EventDate EventCode) : gen foll_EventCode=EventCode[_n+1]

lab var foll_EventCode "Following event"

label define eventlab 1 "ENU" 2 "BTH" 3 "IMG" 4 "OMG" 5 "EXT" 6 "ENT" ///

7 "DTH" 9 "OBE" 10 "DLV" 18 "OBS" 19 "OBL" 20 "1Jan" 21 "NewAgeGroup", modify

lab val foll_EventCode eventlab

tab EventCode foll_EventCode, missing

If no inconsistencies, save the compressed child file:

compress

save child, replace

### Step 2: Create for each child a file with all the events experienced by parents

Assume that for each individual we have the father identification number (FatherId) and mother identification number (MotherId) if they have experienced at least one episode of residence within the HDSS. With these parents’ IDs one can merge the events experienced by parents with the core residency file of the child. In the following, the procedure is explained for mother’s events and has to be replicated for the father’s events if FatherId is available for each child. It is also possible that FatherId is not available or that the only available IDs are that of men related to the mother through union (cohabitation or marriage). In that case FatherId may be replaced by PartnerId and treated the same way, i.e. assuming that the spouse is the main male caretaker of the child. In that case, there may be more than one relevant PartnerId since the mother can have several companions or spouses over the child’s lifetime. In both cases the migration, separation, and death of the father or mother’s partner should be taken into account.

To merge a mother’s and child’s events a file with child and mother IDs must first be created. This file could be based on the child.dta file created in Step 1 if the variable MotherId was already included or it could rely on another specific file containing child and mother IDs:

use child, clear

keep IndividualId MotherId DoB

duplicate drop

Reshape the file in wide format with mothers and their children, to count and rank of the children according to their dates of birth

rename IndividualId ChildId

rename MotherId IndividualId

sort IndividualId

bysort IndividualId (DoB): gen child_rank = _n

reshape wide ChildId DoB, i(IndividualId) j(child_rank)

save mother_children, replace

This mother_children file contains the ID of mothers only together with their children’s ID and date of birth. Then, this mother_children file is merged with the core residency file to get all the events (migration, death…) that the mothers experienced within the HDSS:

use residency, clear

sort IndividualId

merge m:1 IndividualId using mother_children.dta

Dates of birth of children are not required in this file

drop DoB*

rename _merge motherdata

drop if motherdata==1

lab def motherdata 2 "not resident" 3 "matched", modify

lab val motherdata motherdata

There may also be some children whose mothers have never experienced an episode of residence in the HDSS, like mothers who migrated out and never returned before the right-censoring date. In that case motherdata is equal to 2 "not resident" (the mother is not in the core residency file, there is no recorded event for the mother).

For non-resident mothers we need to create an OBE date

replace EventDate=clock("01Jan 2015","DMY")if motherdata==2

replace EventCode=9 if motherdata==2

rename residence residenceMO

save mother, replace

Because the mother file contains the information on the children, it can be reshaped back into a child file, this time with all the mother’s events.

The IndividualId is used to reshape to long format. If IndividualId it is in string format, the following code applies:

bysort IndividualId (EventDate): gen IndividualId_ep = IndividualId+string(_n)

Alternatively if IndividualId is in numerical format:

bysort IndividualId (EventDate): ///

gen IndividualId_ep=string(IndividualId)+string(_n)

Then to reshape in long format:

reshape long ChildId, i(IndividualId_ep) j(child_rank)

capture drop if ChildId == "" //if ChildId is in string format

capture drop if ChildId == . //if ChildId is in numeric format

drop IndividualId_ep

drop Sex

All relevant variables related to the mother should be renamed.

rename EventCode EventCodeMO

rename EventDate EventDateMO

rename Death_Cause Death_CauseMO

All relevant variables related to the child should also be renamed.

rename IndividualId MotherId

rename ChildId IndividualId

order IndividualId EventDateMO EventCodeMO

sort IndividualId EventDateMO EventCodeMO

save childMO, replace

This new file might already be right-censored at the same date as the child file. However, if it is not, the file must be right-censored at the same date as in the child file following instructions from Step 1 (here without explanatory notes) to meet the requirement for Step 3:

sort IndividualId EventDateMO EventCodeMO

expand=2 if IndividualId!=IndividualId[_n+1]& EventDateMO<clock("01Jan 2015","DMY"), gen(duplicate)

sort IndividualId EventDateMO EventCodeMO duplicate

quietly by IndividualId: replace EventDateMO=clock("01Jan 2015","DMY") ///

if duplicate==1

drop duplicate

capture drop lastrecord

quietly by IndividualId: gen lastrecord=(_n==_N)

stset EventDateMO, id(IndividualId) failure(lastrecord==1) exit(time .)

capture drop calendar

stsplit calendar, at(clock("01Jan 2015","DMY")) trim

drop lastrecord

drop if calendar!=0

duplicate drop

sort IndividualId EventDateMO EventCodeMO

quietly by IndividualId : replace EventCodeMO=9 if _n==_N

quietly by IndividualId : replace EventDateMO= clock("01Jan 2015","DMY") ///

if _n==_N

capture drop foll_EventCodeMO

bysort IndividualId (EventDateMO EventCodeMO) : ///

gen foll_EventCodeMO=EventCodeMO[_n+1]

lab var foll_EventCodeMO "Following event"

label define eventlab 1 "ENU" 2 "BTH" 3 "IMG" 4 "OMG" 5 "EXT" 6 "ENT" ///

7 "DTH" 9 "OBE" 10 "DLV" 18 "OBS" 19 "OBL" 20 "1Jan" 21 "NewAgeGroup", modify

lab val foll_EventCodeMO eventlab

tab EventCodeMO foll_EventCodeMO, missing

compress

save childMO, replace

### Step 3: Merge mother’s events with child’s events

In order to use the tmerge program to merge the two files according to child ID and time, all the children represented in the child.dta file must also be represented in the child_mother.dta file. In principle the procedure from Step 2 ensures that this is the case. If there is only one small error, then the tmerge program will stop with an error message.

Below the procedure is explained for the mother’s events:

clear

capture erase child_mother

tmerge IndividualId child(EventDate) childMO(EventDateMO) ///

child_mother(EventDate_final)

format EventDate_final %tc

drop EventDate EventDateMO

rename EventDate_final EventDate

The variable EventCode has to be recoded for episodes that are mother-related events (so that looking at the child’s event’s we can identify where there are mother’s events). Here we chose recode to 18, but any unique value (excluding those already identifying events) can be used:

replace EventCode = 18 if _File==2

order IndividualId EventDate EventCode

sort IndividualId EventDate EventCode

save child_mother, replace

This is exactly the same procedure for the father’s or partner’s events:

clear

capture erase child_mother_partner

tmerge IndividualId child_mother(EventDate) child_PA(EventDate_PA) ///

child_mother_partner(EventDate_final)

format EventDate_final %tc

drop EventDate EventDate_PA

rename EventDate_final EventDate

replace EventCode = 18 if _File==2

order IndividualId EventDate EventCode

sort IndividualId EventDate EventCode

save child_mother_partner, replace

## Stata code for merging child events with siblings’ events

It is also possible to add the events of siblings to the files (in addition to parent’s events). The mother_children.dta file was already created in the previous section and directly used in this section. Using the birth order of each sibling, the younger sibling is defined as the child who precedes the Ego child (identified by IndividualId) and the older sibling as the child who immediately follows Ego. Twins have to be handled carefully.

**Several steps** are necessary to generate the child_relatives file:

- Step 1: Define the sibling’s true rank
- Step 2: Create files for the twin sibling, and the younger and older siblings
- Step 3: Merge the younger and older sibling files with the child file

### Step 1: Define the sibling’s true rank

The child_mother_partner is merged with the mother_children file to identify the rank of Ego among siblings:

use child_mother_partner, clear

keep IndividualId MotherId

duplicate drop

rename IndividualId EgoId

rename MotherId IndividualId

merge m:1 IndividualId using mother_children.dta

drop _merge

The file is reshaped into long format (one sibling per record identified by ChildId):

reshape long ChildId DoB, i(EgoId IndividualId) j(child_rank)

drop if ChildId == ""

The Ego child is identified among the siblings by the individual identifier using an indicator variable:

gen Ego= EgoId==ChildId

rename IndividualId MotherId

sort EgoId DoB

The rank() function is used to determine the birth order of children born of the same mother:

bysort EgoId (DoB) : egen true_child_rank = rank(DoB)

The rank() function gives decimal values to children who were born on the same date (twins). That is if twins are given the same date of birth. Moreover, twins should have the same older or younger sibling and conversely older or younger twin siblings should have the same rank.

replace true_child_rank=int(true_child_rank)

The rank of the Ego child is identified using the indicator variable for Ego:

bysort EgoId (DoB) : egen Ego_rank = max(cond(Ego==1,true_child_rank,0))

save child_mother_Ego, replace

### Step 2: Create files for the twin sibling, and the younger and older siblings

Select the twin siblings:

use child_mother_Ego, clear

bysort EgoId (child_rank) : keep if true_child_rank==Ego_rank & ChildId!= EgoId

keep ChildId

duplicates drop

rename ChildId IndividualId

sort IndividualId

save twin, replace

Select the non-twin siblings:

use child_mother_Ego, clear

bysort EgoId (child_rank) : drop if true_child_rank==Ego_rank & ChildId!= EgoId

keep ChildId

duplicates drop

rename ChildId IndividualId

sort IndividualId

save non_twin, replace

Merge the file for twins with the core residency file to get their event history:

use residency, clear

sort IndividualId

merge m:1 IndividualId using twin.dta

keep if _merge==3

drop _merge

Rename all the relevant variables:

rename EventDate EventDateTwin

rename EventCode EventCodeTwin

rename Sex SexTwin

rename DoB DoBTwin

rename residence residenceTwin

rename Death_Cause Death_CauseTwin

Delete non relevant variables e.g.:

drop datebeg

Append the twin with the non-twin file:

append using non_twin

Recode OBE for non-twins

recode EventDateTwin .= clock("01Jan 2015","DMY")

recode EventCodeTwin .=9

sort IndividualId EventDateTwin

save twin, replace

erase non_twin.dta

Select the younger siblings (including twin younger siblings):

use child_mother_Ego, clear

bysort EgoId (child_rank) : keep if true_child_rank==Ego_rank+1

keep ChildId

duplicates drop

rename ChildId IndividualId

sort IndividualId

save ysibling, replace

Merge the file of younger siblings with the core residency file to get their event history:

use residency, clear

sort IndividualId

merge m:1 IndividualId using ysibling.dta

keep if _merge==3

drop _merge

Rename all the relevant variables:

rename IndividualId YsiblingId

rename EventDate EventDateYsibling

rename EventCode EventCodeYsibling

rename Sex SexYsibling

rename DoB DoBYsibling

rename residence residenceYsibling

rename Death_Cause Death_CauseYsibling

drop datebeg

save ysibling, replace

The same is done for the older siblings. Select the older siblings (including twin older siblings), and merge the older sibling’s file with the core residency file to get their event history (here without explanatory notes):

use child_mother_Ego, clear

bysort EgoId (child_rank) : keep if true_child_rank==Ego_rank-1

keep ChildId

duplicates drop

rename ChildId IndividualId

sort IndividualId

save osibling, replace

use residency, clear

sort IndividualId

merge m:1 IndividualId using osibling.dta

keep if _merge==3

drop _merge

rename IndividualId OsiblingId

rename EventDate EventDateOsibling

rename EventCode EventCodeOsibling

rename Sex SexOsibling

rename DoB DoBOsibling

rename residence residenceOsibling

rename Death_Cause Death_CauseOsibling

drop datebeg

save osibling, replace

### Step 3: Merge the younger and older sibling files with the child’s file

Merge the twins file with the child file that already includes parents’ history:

clear

capture erase child_mother_partner_twin

tmerge IndividualId child_mother_partner(EventDate) twin(EventDateTwin) ///

child_mother_partner_twin(EventDate_final)

format EventDate_final %tc

drop EventDate EventDateTwin

rename EventDate_final EventDate

replace EventCode = 18 if _File==2

order IndividualId EventDate EventCode

sort IndividualId EventDate EventCode

save child_mother_partner_twin, replace

Merge the younger sibling’s file with the child’s file that already includes parents’ and twin’s history:

clear

capture erase child_mother_partner_t_y

tmerge IndividualId child_mother_partner_twin(EventDate)ysibling(EventDateY) ///

child_mother_partner_t_y(EventDate_final)

format EventDate_final %tc

drop EventDate EventDateY

rename EventDate_final EventDate

replace EventCode = 18 if _File==2

order IndividualId EventDate EventCode

sort IndividualId EventDate EventCode

save child_mother_partner_t_y, replace

Merge the older sibling’s file with the children file that includes parents’ and younger siblings’ histories:

clear

capture erase child_mother_partner_sibling

tmerge IndividualId child_mother_partner_t_y (EventDate)osibling(EventDateO) ///

child_mother_partner_sibling(EventDate_final)

format EventDate_final %tc

drop EventDate EventDateO

rename EventDate_final EventDate

replace EventCode = 18 if _File==2

order IndividualId EventDate EventCode

sort IndividualId EventDate EventCode

save child_mother_partner_sibling, replace

### Step 4: Optional: Restricting observations to under-5 year old

In order to analyse under-five year old mortality for example, we should restrict the file to this age group. This involves creating an extra record corresponding to the child’s 5th birthday:

use child_mother_partner_sibling, clear

capture drop censor_death

gen censor_death=(EventCode==7) if residence==1

capture drop datebeg

bysort IndividualId (EventDate): ///

gen double datebeg=cond(_n==1,DoB,EventDate[_n-1])

stset EventDate if residence==1, id(IndividualId) failure(censor_death==1) ///

time0(datebeg) origin(time DoB) exit(time .)

capture drop fifthbirthday

* 157788000000 gives exact birthday in %20.0f (dis %20.0f 5*365.25*24*60*60*1000)

stsplit fifthbirthday, at(157788000000) trim

drop lastrecord

drop if fifthbirthday!=0

duplicate drop

save child_mother_partner_sibling, replace

# **Caveats and limitations**

## Caveats related to HDSS data collection

HDSS data are typically collected from households at least once a year following an initial census of the population within a geographically delimited area. Since households (and the individuals in them) move (migrate), dissolve (die) or are created (born), at each round of data collection, interviewers need to update all this information. Some households may move entirely within the surveillance site, so that only their location needs updating but the household membership remains the same. Some households may leave the region altogether and the members “lost-to-follow up”. In such circumstances, interviewers will ask neighbours about what happened to the household to ensure that no members remained in the site (or are found in a new location within site). Likewise, a new household may enter the site, following baseline, will be added to the surveillance records. A new household ID is assigned, and each member an individual ID. In cases when a member leaves a household, her individual ID is maintained. These dynamics are carefully recorded and checked. However, it is possible that some misunderstandings of who moved where, or who belongs to which household may arise, particularly when update rounds are infrequent. There is also a potential bias due to both left and right censoring, if the population leaving/ entering surveillance have specific and different characteristics to those who do not move. It should be kept in mind that some households are tracked for shorter periods than others, and not the entire duration of HDSS surveillance. Using event history analysis, which account for attrition, with the correct exposure time, it is possible to partly address such bias. Figure 1 provides an example of how we can still use the censored data.

A further related caveat relates to defining household membership. In the above methods, we rely on shared residence, using a de facto definition. However, it is possible that the HDSS itself defines residence differently. For example, an individual who works in an area outside of the surveillance site, but sends remittances, or visits once a month may be considered a household member by an HDSS that uses a de jure definition. In our analysis we rely on data from the Farafenni HDSS (in the Gambia), which uses a de facto definition of household membership (living in same house and sharing the same cooking arrangements). We advise caution when working with HDSS sites (or other sources of longitudinal data) that use a de jure definition. Although the same methods can be used, the interpretation of the results may differ.

## Limitations related to the methodology

The methods we lay out above (to construct time-varying household structure and composition) rely on accurate recording of household and individual identifiers and relationship to parents. The detail and intensity of data collection from HDSS [6] suggests that there should not be a problem with the quality of these identifiers. However, in some contexts this information may be inaccurate. Two examples of misidentification of parents illuminate why using parent IDs could be inaccurate: 1) In contexts where child fostering is common, it is common for foster-parents to identify as true parents, even if they may be a distant relative of the child, or not blood related. 2) In contexts where sexual partnering is fluid, and it is not possible to identify the father. In both of these cases, the “true” composition of the household may not be reached, though the structure remains the same (since kin relations are not needed in this case). The first context of high levels of fostering is likely not to lead to incorrect identification since HDSS also cover pregnancies. Children born after the time of baseline data collection are firstly recorded as pregnancies, and at a minimum are directly linked to their mothers. Thus, at least when interested in young children, we can reliably link them to their mothers. We recommend employing the methods described above linking kin, using reliable data where possible, and when in contexts of potentially inaccurate links, to acknowledge the limitations of this method.

**References**

1. Bocquier P, Ginsburg C, Herbst K, Sankoh OA, Collinson MA. Manual of Event History Data Analysis using Health and Demographic Surveillance System Data. BMC Res Notes. 2017;10:1–3.

2. Bocquier P, Ginsburg C, Collinson MA. A training manual for event history analysis using longitudinal data. BMC Res Notes. 2019;12:1–5.

3. INDEPTH. INDEPTH i-Share Data Repository. 2017. http://www.indepth-ishare.org/index.php/home.

4. Menashe-Oren A, Bocquier P, Ginsburg C, Compaoré Y, Collinson MA. The dynamic role of household structure on under-5 mortality in southern and eastern sub-Saharan Africa. Demogr Res. 2023;49 Article 11:249–94.

5. Bocquier P, Ginsburg C, Menashe-Oren A, Compaoré Y, Collinson M. The Crucial Role of Mothers and Siblings in Child Survival: Evidence From 29 Health and Demographic Surveillance Systems in Sub-Saharan Africa. Demography. 2021;58:1687–713.

6. Herbst K, Juvekar S, Jasseh M, Berhane Y, Chuc NTK, Seeley J, et al. Health and demographic surveillance systems in low- and middle-income countries: history, state of the art and future prospects. Glob Health Action. 2021;14:1974676.
